# Coordinated Electronic‐Transport Regulation and Multiscale Phonon Scattering in Sb–La Co‐Doped GeTe

**DOI:** 10.1002/advs.77821

**Published:** 2026-09-27

**Authors:** Ting Lu, Huangshui Ma, Mingquan Li, Min Hong

**Affiliations:** ^1^ Centre for Future Materials School of Science Engineering and Digital Technologies University of Southern Queensland Springfield Australia

**Keywords:** carrier concentration, GeTe, La doping, phonon scattering, Sb doping, thermoelectric performance

## Abstract

GeTe is a promising p‐type thermoelectric material for mid‐to‐high temperature applications because of its favorable electronic structure and excellent transport potential. However, its thermoelectric performance is still limited by excessive hole concentration and insufficient phonon scattering. Herein, rare‐earth La was co‐doped with Sb into GeTe via a melting–annealing–spark plasma sintering process, and the microstructure and thermoelectric transport properties of the samples were systematically evaluated. The thermoelectric characterization reveals that the co‐doping strategy significantly lowers the thermal conductivity without compromising electrical performance, thereby contributing to enhanced thermoelectric performance. The incorporation of Sb effectively regulates the hole concentration, preserves the electrical transport properties, and reduces the electronic thermal conductivity. In addition, La doping induces strain‐field fluctuations and local structural disorder, introducing multiscale micro‐ and nano‐structural features, which significantly strengthen phonon scattering and thereby suppress the lattice thermal conductivity. As a result, the Ge_0.89_Sb_0.1_La_0.01_Te composition achieves a peak *zT* of 2.8 at 793 K. These results demonstrate that rare earth‐assisted composition tuning is an effective strategy for improving the thermoelectric performance of GeTe‐based materials.

## Graphical abstract

Synergistic Sb–La co‐doping simultaneously regulates hole concentration and creates strain‐field fluctuations and multiscale structural disorder in GeTe. Electrical transport is preserved, while electronic and lattice thermal conductivities are strongly suppressed. Consequently, thermoelectric figure of merit values have been enhanced, highlighting rare‐earth‐assisted composition tuning as a powerful route to high‐performance GeTe thermoelectrics.

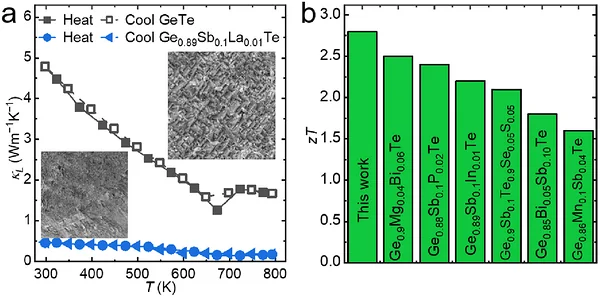

## 1 Introduction

The growing need for clean energy has spurred considerable interest in energy conversion technologies that can harvest and utilize energy more sustainably [1, 2, 3]. Thermoelectric technology, which directly converts heat into electricity and vice versa via the Seebeck and Peltier effects, is regarded as a promising clean‐energy technology [4, 5]. In the power‐generation mode, thermoelectric devices can convert waste heat directly into electricity, offering opportunities for energy harvesting in automotive and industrial systems, space power generation, and self‐powered wearable electronics [6, 7, 8]. Conversely, in the electricity‐to‐heat mode, thermoelectric devices convert electrical energy into heat through the Peltier effect, enabling precise and rapid thermal control without moving parts [9, 10, 11, 12]. The broad applicability of thermoelectric technology in both power‐generation and cooling applications is largely associated with the intrinsic advantages of thermoelectric devices, including their simple structural design, reliable operation, low noise levels, and minimal maintenance requirements [13]. However, the practical implementation of thermoelectric technology is still constrained by its relatively low conversion efficiency and the high cost associated with materials and device fabrication, both of which limit its wider application [14].

The thermoelectric performance of a material is commonly evaluated by the dimensionless figure of merit, *zT*, defined as

(1) $$zT=S^{2}\sigma T/\kappa$$

where *S* is the Seebeck coefficient, *σ* is the electrical conductivity, *κ* denotes the total thermal conductivity, and *T* is the absolute temperature [15]. The total thermal conductivity can be further expressed as *κ* = *κ_L_* + *κ_e_*, where *κ_L_* and *κ_e_* denote the lattice and electronic contributions, respectively. *S* ^2^ *σ*, namely the power factor (*PF*), represents the electronic contribution to thermoelectric performance, whereas a smaller *κ* is generally favorable for improving *zT* [16].

However, these TE parameters are often coupled with each other. Typically, increasing carrier concentration may improve *σ* but can simultaneously suppress *S* [17]. Lowering *κ_L_* through nanostructuring or defect engineering may also affect the charge carrier transport [18]. Therefore, optimizing thermoelectric materials requires a careful balance among high *S*, high *σ*, and low *κ*, and managing these competing effects remains a central issue in thermoelectric research [19].

Thermoelectric materials exhibit their maximum performance within different temperature ranges, which determines their suitability for various application scenarios. For example, Bi_2_Te_3_‐based materials [20, 21] typically demonstrate superior thermoelectric performance near room temperature and are therefore widely employed in commercial thermoelectric devices. In the low‐temperature range, Te‐free thermoelectric materials have recently attracted considerable attention as promising alternatives to conventional Te‐containing materials, particularly for low‐grade heat harvesting. In contrast, Half‐Heusler alloys [22, 23], skutterudites [24, 25], and several oxide‐based thermoelectric materials generally operate at elevated temperatures, making them attractive for industrial waste‐heat recovery and space power‐generation applications. In the intermediate temperature range, PbTe [26, 27], GeTe [28, 29], SnSe [30, 31], and argyrodite‐based compounds [32, 33, 34] have emerged as some of the most promising thermoelectric materials owing to their high thermoelectric performance, rendering them suitable candidates for recovering waste heat from automotive systems and industrial processes.

Among the various mid‐temperature thermoelectric materials, GeTe has emerged as one of the most promising candidates owing to its favorable intrinsic properties and outstanding thermoelectric potential [35, 36]. GeTe undergoes a phase transition from rhombohedral (R3m) to cubic structures (Fm‐3m) at about 700 K [37]. In addition, the presence of intrinsic Ge vacancies, together with various microstructural features such as grain boundaries, stacking faults, and point defects, provides considerable flexibility for tuning both electronic and thermal transport properties [38]. These characteristics have enabled GeTe to achieve thermoelectric performance comparable to or even exceeding that of other well‐known mid‐temperature thermoelectric materials, including PbTe and SnSe [39, 40].

However, pristine GeTe generally exhibits an excessively high hole concentration originating from Ge vacancies, which limits the optimization of its electrical transport properties. To address this issue, extensive research efforts have focused on vacancy engineering and carrier concentration regulation through elemental doping. In particular, aliovalent dopants such as Bi [40, 41] and Sb [42, 43] have been widely employed to optimize carrier transport and improve *PF*. Nevertheless, individually doped GeTe compounds often retain a relatively high *κ_L_*. Consequently, various co‐doping strategies [44, 45, 46] have been developed to simultaneously regulate carrier transport and enhance phonon scattering, leading to further reductions in *κ_L_* [47]. Rare‐earth doping in GeTe has been reported to significantly reduce *κ_L_* by inducing lattice fluctuations and to effectively modulate charge carrier transport [48, 49, 50, 51]. For example, Sc–Bi co‐doping has been demonstrated to optimize the carrier concentration while simultaneously reducing κ*_L_* through enhanced phonon scattering, resulting in improved thermoelectric performance [52]. In addition, Y doping has been shown to substantially reduce the carrier concentration of GeTe and consequently optimize its electrical transport properties [53]. These studies highlight the effectiveness of rare‐earth incorporation in tailoring the electronic and lattice transport properties of GeTe, while also suggesting that the resulting effects are highly dependent on the identity and concentration of the dopant. Therefore, we investigate Sb, La co‐doped GeTe to determine whether La doping produces similar changes in carrier transport or leads to different changes in the electronic structure and lattice dynamics compared with previously reported rare‐earth dopants.

**FIGURE 1:**
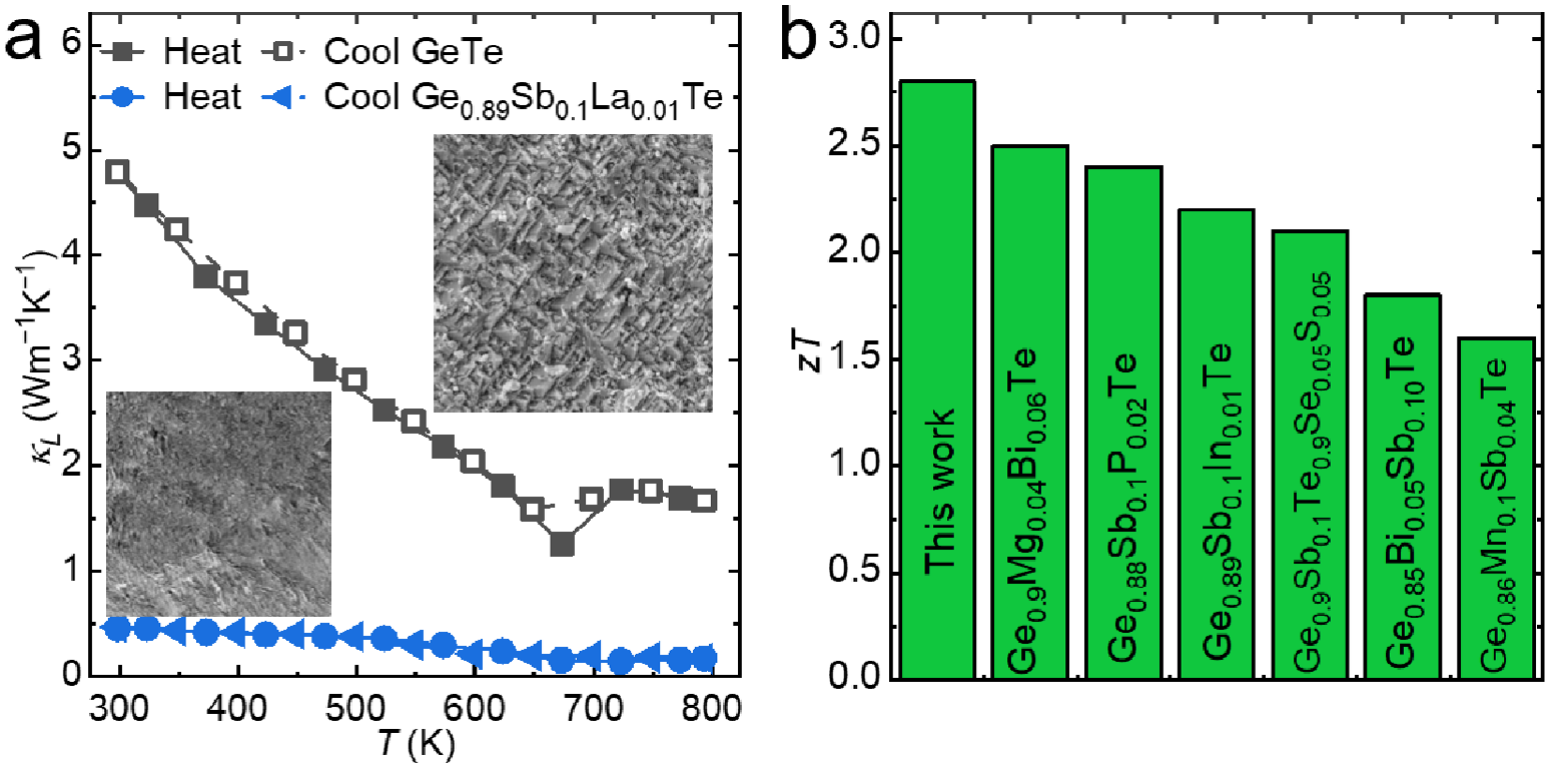
Overview of the conceptual framework of enhancing thermoelectric performance. (a) Comparison of *κ_L_* between GeTe and Ge_0.89_Sb_0.1_La_0.01_Te. Insets are micro and nanostructures of GST‐La (b) Comparison of pesk *zT* between GST‐La and other GeTe materials [46, 54, 55, 56, 57].

In this work, rare‐earth co‐doped GeTe samples were synthesized via a conventional melting‐ annealing‐ spark plasma sintering (SPS) method. The Ge_0.89_Sb_0.1_La_0.01_Te (GST‐La) sample achieved a *zT* value of 2.8 at 793 K, which is primarily attributed to the substantial reduction in *κ* while maintaining a favorable *PF*. Sb doping effectively regulates the hole concentration, thereby optimizing the electrical transport properties and reducing *κ_e_*. In addition, La doping enhances lattice disorder and introduces multiple microstructural features, which further suppress *κ_L_*. The relatively low *κ_L_* may arise from multiple phonon‐scattering mechanisms, including point defects associated with the increasing lattice disorder, domain boundaries, planar defects, ridge‐like structures, and grain boundaries. Collectively, these structural features provide a basis for the improved thermoelectric performance of GST‐La (Figure 1).

## 2 Results and Discussion

**FIGURE 2:**
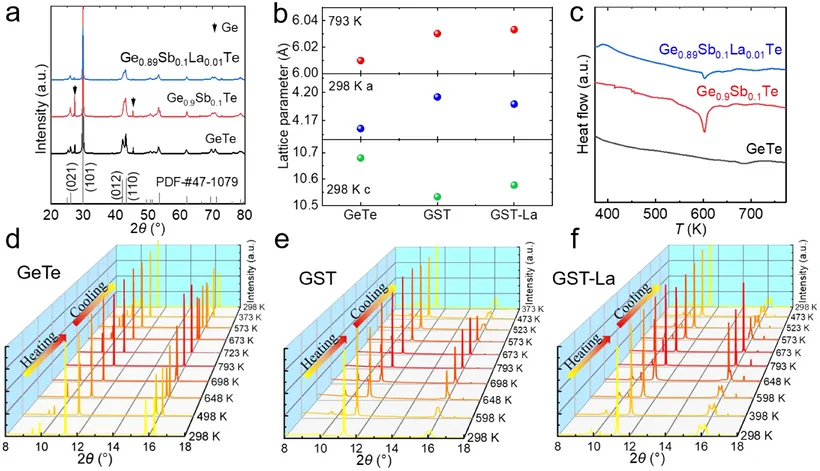
XRD, PD, and DSC characterization of pristine and doped GeTe samples. (a) XRD patterns of pristine and doped GeTe samples. (b) Lattice parameters of pristine and doped GeTe samples at 298 and 793 K. (c) DSC curves of pristine and doped GeTe samples. (d), (e), and (f) are Synchrotron radiation powder diffraction patterns for GeTe, GST, and GST‐La, respectively.

Figure 2a presents the X‐ray diffraction (XRD) patterns of the GeTe, Ge_0.9_Sb_0.1_Te (GST), and Ge_0.89_Sb_0.1_La_0.01_Te (GST‐La) powder samples. The diffraction peaks can be indexed as the R3m phase with the standard XRD pattern shown in the bottom [58]. The diffraction peaks at approximately 27.3° and 45.4°, corresponding to Ge (111) and (220), respectively, confirm the presence of elemental Ge precipitates in the GST sample. The pronounced Ge precipitation may be related to the prolonged annealing during synthesis. Since the formation energy of Ge vacancies is lower in the cubic than in the R3m phase, prolonged annealing may promote defect redistribution and Ge segregation, thereby facilitating Ge precipitation. Compared with the GST sample, the GST‐La sample exhibits a reduced Ge impurity signal in the XRD pattern. This observation suggests that La doping might influence the defect chemistry associated with Ge vacancies and contribute to improved compositional homogeneity within the material. In addition, the diffraction peaks associated with GeTe are markedly suppressed in the GST‐La sample, exhibiting intensities less than half of those in the GST sample. Since the two samples were synthesized from the same batch and the reduction occurs consistently across the diffraction peaks, as is also observed in the powder diffraction (PD), this intensity decrease is unlikely to arise from differences in sample preparation or a specific preferred orientation. The reduced overall diffraction intensity suggests a change in the structural order of GST‐La and may be associated with an increased degree of local structural disorder. This interpretation may be consistent with the enthalpy‐relaxation feature observed in the differential scanning calorimetry (DSC) measurement. Such locally disordered structures may act as additional phonon‐scattering centers and contribute to the reduced *κ*_L_.

Figure 2d–f display the normalized temperature‐dependent synchrotron radiation powder diffraction patterns of the GST‐La sample during the heating and cooling processes from room temperature to 793 K. No additional diffraction peaks are observed throughout the thermal cycling process, indicating that the phase remains stable within the investigated temperature range. During heating, the structural transition from the rhombohedral phase to the cubic phase occurs between 598 and 648 K, as evidenced by the evolution of the diffraction patterns. Additionally, the intensity of synchrotron radiation powder diffraction for the GST‐La sample also has a markly decrease over half than that of the GST sample, confirming the result of XRD.

The phase transition behavior is further supported by the DSC results shown in Figure 2c. The DSC curve reveals a phase‐transition temperature of approximately 600 K for the GST‐La sample, which is similar to that observed for the undoped GST sample. Therefore, the introduction of La does not appear to cause a significant change in the phase‐transition temperature. In contrast, previous studies have reported that Mn doping substantially reduces the phase‐transition temperature [59], whereas La doping has little effect. This difference may arise from the distinct interactions of the dopants with the host lattice. In the present GST‐La system, the relatively low La concentration may be insufficient to significantly perturb the lattice distortion and structural stability, resulting in a nearly unchanged phase‐transition temperature. Additionally, an exothermic peak centered at approximately 400 K is observed for the GST‐La sample, which may be associated with the enthalpy relaxation of locally disordered or metastable structures. The concurrent decrease in overall diffraction intensity suggests structural rearrangement upon heating, potentially involving the relaxation of metastable configurations [60, 61].

Figure 2b presents the lattice parameters derived from the PD results at room temperature (RT) and 793 K. At room temperature, the rhombohedral (R3m) phase exhibits lattice parameters a of 4.162 Å for GeTe and 4.195 Å for GST, while GST‐La shows an intermediate value of 4.186 Å, slightly smaller than that of GST. The lattice parameters c are 10.680 Å for GeTe, 10.533 Å for GST, and 10.577 Å for GST‐La, respectively. At 793 K, after GeTe transforms to the cubic phase, the lattice parameter of GST‐La is 6.033 Å, slightly larger than that of GST (6.030 Å). La possesses a substantially larger atomic mass and ionic radius (La^3+^, ∼1.16 Å) than Ge (Ge^2+^, 0.73 Å). Consequently, the substitution of Ge by La introduces pronounced strain‐field fluctuations and local structural distortions, which perturb the rhombohedral R3m lattice and lead to a contraction of the *a*‐axis accompanied by an expansion of the *c*‐axis [62].

**FIGURE 3:**
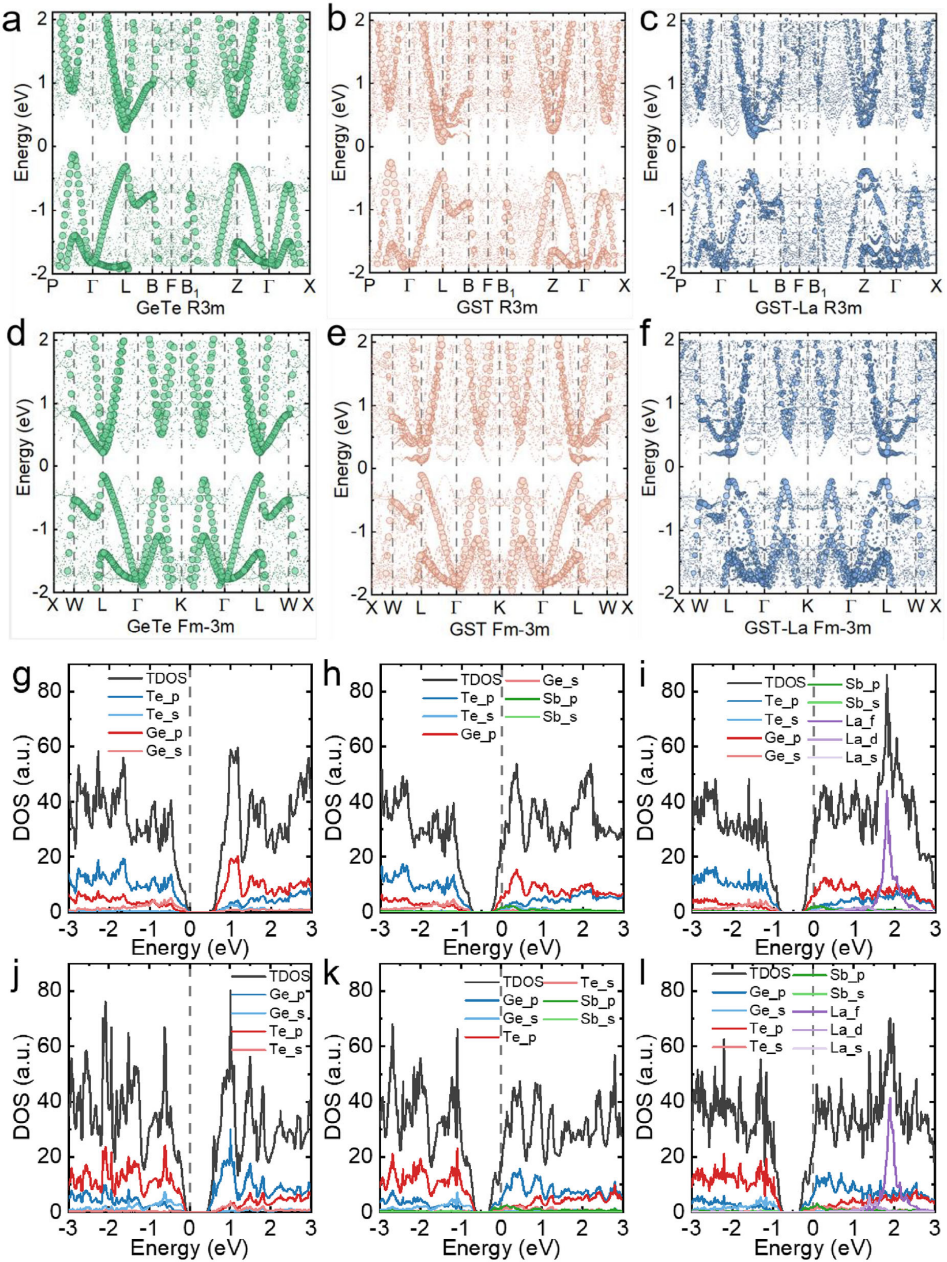
DFT‐calculated band structures and DOS at RT and high temperature. DFT‐calculated flat band structures of R3m (a) Ge_27_Te_27_, (b) Ge_24_Sb_3_Te_27,_ and (c) Ge_23_Sb_3_La_1_Te_27_ at RT. DFT‐calculated flat band structures of cubic (d) Ge_27_Te_27_, (e) Ge_24_Sb_3_Te_27,_ and (f) Ge_23_Sb_3_La_1_Te_27_ at high temperature. DFT‐calculated DOS maps of R3m g) Ge_27_Te_27_, (h) Ge_24_Sb_3_Te_27,_ and (i) Ge_23_Sb_3_La_1_Te_27_ at RT. DFT‐calculated flat band structures of cubic (j) Ge_27_Te_27_, (k) Ge_24_Sb_3_Te_27,_ and (l) Ge_23_Sb_3_La_1_Te_27_ at high temperature.

Figure 3a–c present the density functional theory (DFT) calculated band structures of rhombohedral Ge_27_Te_27_, Ge_24_Sb_3_Te_27,_ and Ge_23_Sb_3_La_1_Te_27_ at RT. As shown in Figure 3a, pristine R3m GeTe exhibits an indirect band gap of 0.40 eV, with the valence‐band maximum (VBM) located along the P–Γ direction and the conduction‐band minimum (CBM) at the L point. Large orbital‐projected bubbles are continuously distributed along the dispersive bands, indicating strong orbital hybridization and highly delocalized electronic states, which relate to strong carrier transport and are therefore favorable for high *σ*. After Sb doping (Figure 3b), the band gap decreases to 0.35 eV. Although the VBM remains dominated by large orbital‐projected bubbles, additional small projected states appear near the CB edge, leading to a reconstruction of the CB edge around the CBM, indicating a redistribution of orbital character in the conduction band. Such band‐edge reconstruction is expected to strengthen the electric transport. With further La doping (Figure 3c), the band gap increases to 0.43 eV. Meanwhile, the orbital‐projected bubbles near the VBM become noticeably smaller than those in Ge_24_Sb_3_Te_27_,, indicating a redistribution of orbital character along VB.

Figure 3d–f shows the calculated band structures of the cubic phase. Cubic Ge_27_Te_27_ exhibits a direct band gap of 0.36 eV at the L point (Figure 3d). After Sb alloying (Figure 3e), the band gap decreases to 0.22 eV. Meanwhile, the energy separation among CBM, the edge near W, and the edge near the K point is reduced, indicating enhanced CB convergence. The emergence of additional low‐energy CB increases the number of accessible electronic states near the CB, which is expected to modify the carrier transport. After further La doping (Figure 3f), the band gap increases to 0.44 eV. In addition, the VBM becomes flatter, and the energy separation between the VB edge along L–Γ and that along Γ–K is reduced, indicating enhanced VB convergence. The increased valley degeneracy is expected to increase the density‐of‐states (DOS) effective mass, which is favorable for maintaining a high *S*. Meanwhile, the enlarged band gap suppresses bipolar excitation at elevated temperatures. Together, these electronic‐structure modifications are consistent with the experimentally observed slight increase in *σ* while preserving a high *PF* at high temperatures.

Figure 3g–i show the calculated DOS of the rhombohedral phase at room temperature. For Ge_27_Te_27_ (Figure 3g), the Fermi level (*E*_f_) is located close to the VBM. After Sb alloying (Figure 3h), the *E*_f_ shifts toward the CB side, accompanied by a redistribution of the electronic states near the band edges and an increased DOS around E_f_. This electronic‐structure evolution corresponds to the experimentally observed reduction in the p‐type *σ*. After further La doping (Figure 3i), the DOS at the *E*_f_ decreases slightly, corresponding to the experimentally observed increase in the p‐type *σ*. In addition, a sharp peak appears at approximately 1.6 eV, which originates predominantly from the localized La‐f orbitals.

Figure 3j is the DOS of cubic GeTe at high temperature, which also has a E_f_ at a bit over VBM with a DOS of approximately 0. Sb doping moves *E*_f_ to the CB and increases the DOS at E_f_. Further La doping increases the DOS at E_f_ and flattens a peak near E_f_, which means electron delocalization. The DOS peak adjacent to the *E*_f_ becomes broader and flatter, suggesting enhanced electronic‐state delocalization and stronger orbital hybridization. Combined with the VB convergence revealed by the band structure, these electronic‐structure modifications help explain the experimentally observed preservation of a high *PF* at elevated temperatures.

Together, these electronic‐structure modifications may contribute to the experimentally observed slight enhancement in *σ* and the retention of a high *PF* in the Sb–La co‐doped sample at elevated temperatures. However, the *σ* is governed not only by the electronic band structure but also by carrier concentration and carrier‐scattering processes. Therefore, the calculated band‐structure results should be regarded as providing qualitative insight into the possible electronic origins of the observed transport behavior, rather than establishing a direct causal relationship. Although the La concentration in the DFT supercell does not exactly reproduce the nominal experimental composition, the calculations nevertheless provide useful qualitative information on the electronic‐structure modifications associated with Sb–La co‐doping.

**FIGURE 4:**
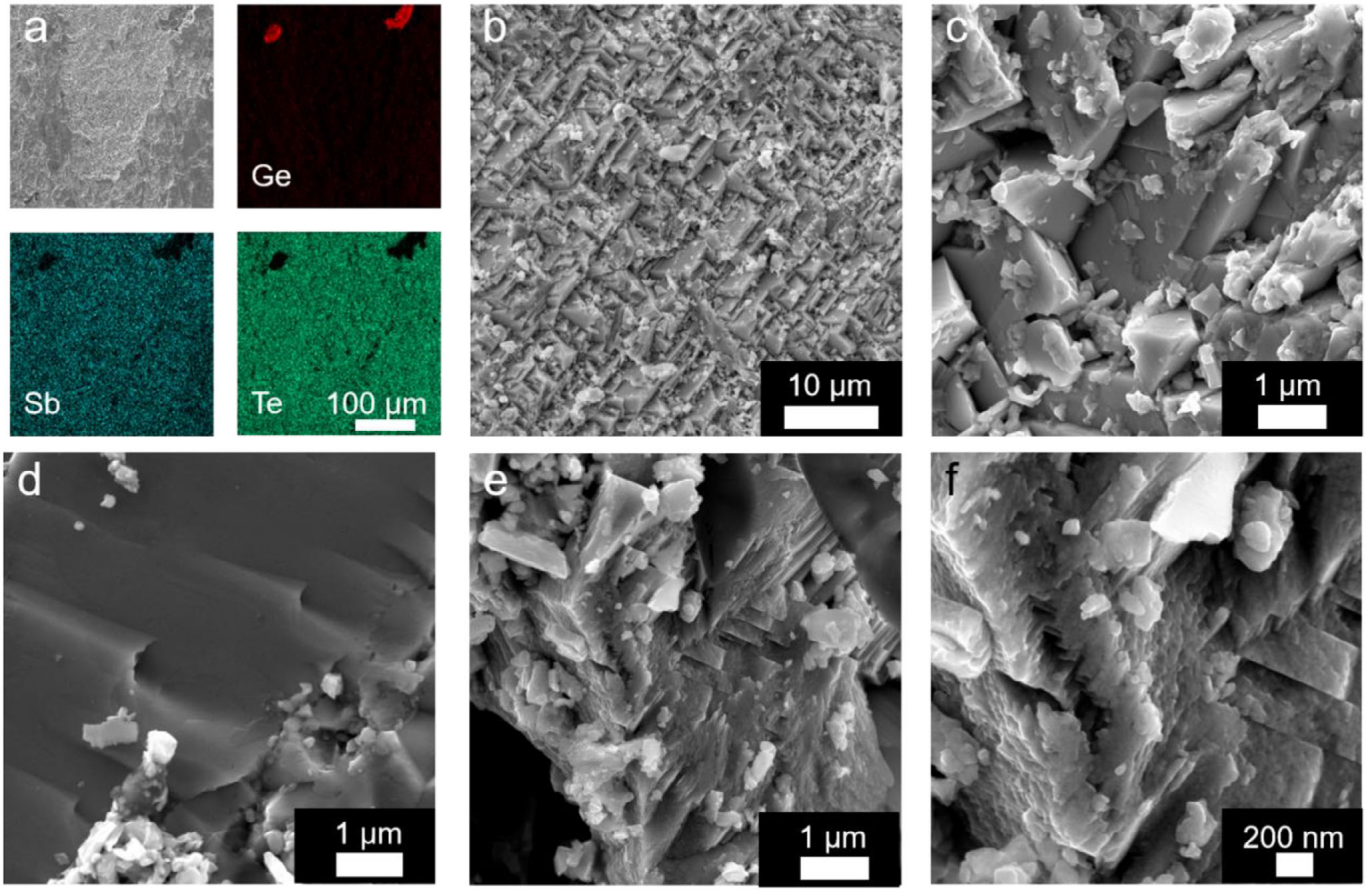
SEM analysis of the GST‐La sample. (a) Low‐magnification SEM image and corresponding EDS elemental mapping of the GST‐La sample. (b) Enlarged view of the fractured region marked in (a). (c) Higher‐magnification image of the region shown in (b). (d) High‐magnification SEM image of the cross‐sectional surface of the GST‐La sample. (e) High‐magnification SEM image of the GST‐La powder. (f) Enlarged view of the region shown in (e).

Figure 4a shows the low‐magnification scanning electron microscopy (SEM) image of the GST‐La sample, which has larger fractured regions than the GeTe and GST samples (Figures S5 and S6). Energy‐dispersive X‐ray spectroscopy (EDS) maps reveal Ge impurities with characteristic sizes of approximately 10–30 µm. La element is difficult to observe in SEM for its low percentage (Tables S2–S4). Figure 4b shows the fractured region, where densely packed crystals are observed. The enlarged image in Figure 4c indicates that these crystals exhibit morphologies with clear facets and are arranged irregularly. To further examine the microstructure, the cross‐sectional surface of the sample was observed, as shown in Figure 4d. The crystals are layered and tilted relative to the SPS pressing direction. Figure 4e,f show SEM images of the ground powder, where layered crystals oriented in different directions can be observed. Such randomly distributed layered structures may contribute to phonon scattering. In addition, nanoscale features with sizes of approximately 50–200 nm are observed, which may further enhance phonon scattering.

**FIGURE 5:**
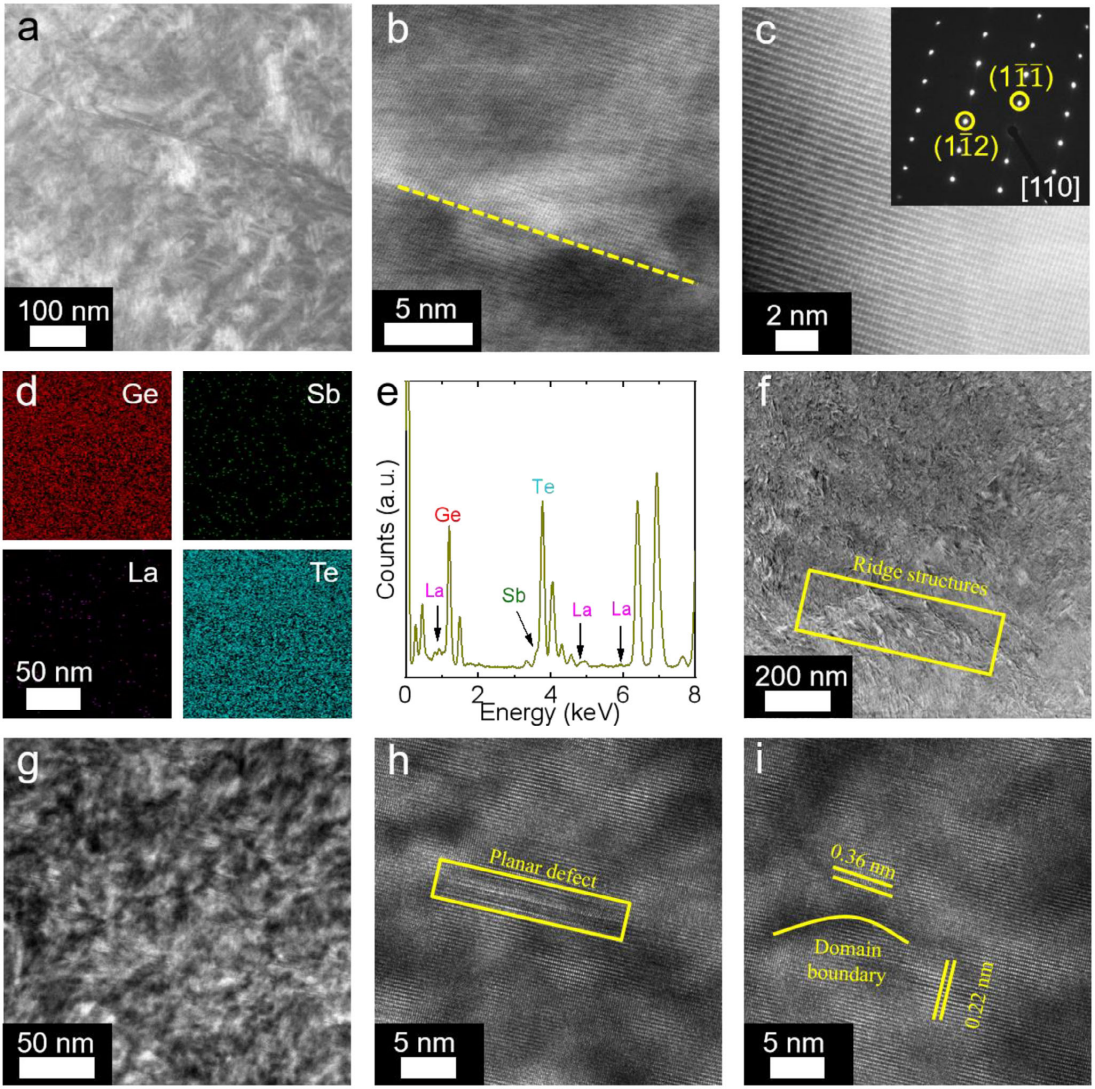
TEM analysis of GST‐La sample. (a) TEM image of a grain boundary. (b) HRTEM image of the grain boundary shown in (a). (c) Atomic‐resolution HAADF‐STEM image of the GST‐La sample, together with the corresponding SAED pattern taken along the [110] zone axis. (d) EDS elemental mapping and (e) EDS spectrum of the region in (a). (f) TEM image showing ridge‐like structures. (g) TEM image of an area containing multiple planar defects. (h) HRTEM image of a planar defect. (i) HRTEM image of domains with different orientations.

Figure 5a,b show the low‐ and high‐magnification transmission electron microscopy (TEM) images of a grain boundary, which can scatter phonons. Figure 5c shows a high‐magnification TEM image and the corresponding selected‐area electron diffraction (SAED) pattern, both of which reveal clear lattice fringes along the [111] direction, indicating high crystallinity. Figure 5d presents the EDS elemental maps corresponding to Figure 5b, showing a homogeneous distribution of the four elements and no detectable nanoscale impurities. Figure 5e shows the EDS spectrum of the GST‐La sample. Although La is present at a low concentration, several characteristic La peaks can still be identified, providing clear evidence for the successful incorporation of La into the GST lattice.

Figure 5f shows a low‐magnification TEM image of the GST‐La sample, in which numerous irregularly distributed ridge‐like structures are observed. Based on previous literature reports [63], these structures have been attributed to the deformation of the herringbone arrangement during annealing, accompanied by the formation and sliding of abundant van der Waals gaps. The stripe‐like reflections in the inset SAED patterns further corroborate the presence of van der Waals (vdW) structures. These vdW gaps correspond to locally ordered Ge‐vacancy arrangements, which can effectively suppress *κ_L_* by enhancing phonon scattering.

Figure 5g shows numerous planar defects, and Figure 5h provides a high‐magnification image of the planar defect marked in Figure 5f. The planar defects are a kind of dislocation, which can significantly scatter phonons. Figure 5i shows lattice spacings of 0.36 and 0.22 nm, corresponding to the [111] and [220] planes, respectively. The coexistence of lattice fringes with different orientations indicates the presence of domains, and the dark regions are attributed to domain or subdomain boundaries. These domains may be associated with the nanoscale features observed in the SEM image shown in Figure 5f. Taken together, the grain and domain boundaries, dislocations associated with van der Waals gaps, planar defects, nanoscale structures, La‐doping‐induced scattering centers, and increased lattice disorder may collectively contribute to the suppression of the *κ_L_*.

**FIGURE 6:**
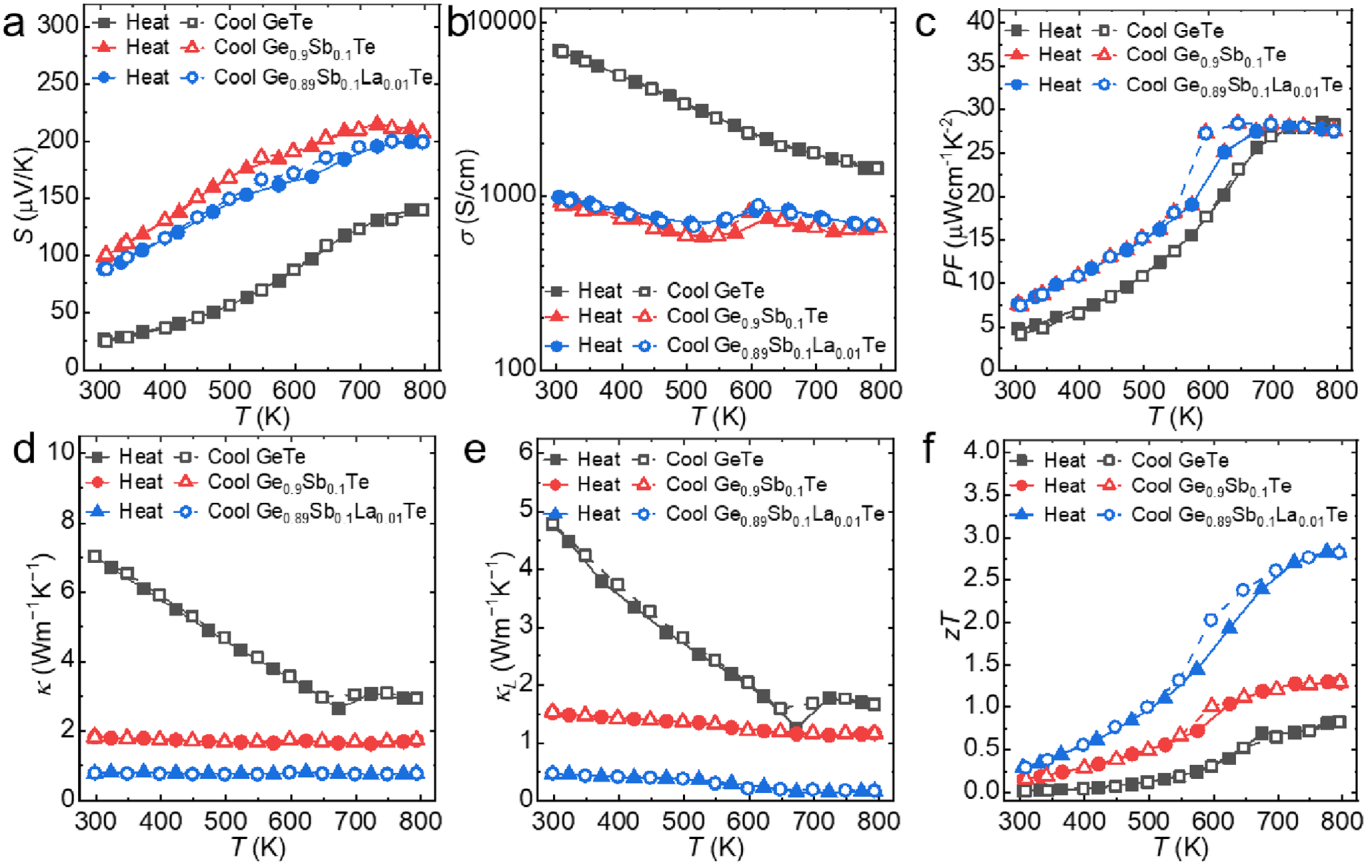
Thermoelectric properties of pristine and doped GeTe samples during heating and cooling cycles. (a) *σ*, (b) *S*, (c) *PF*, (d) *κ*, (e) *κ*_l_, and (f) *zT* as a function of temperature.

Figure 6a shows the temperature‐dependent *S* of the samples. At RT, GST has an *S* of about 100 µV/K, while those of GST‐La and GeTe are about 90 and 25 µV/K, respectively. At 793 K, *S* of GST‐La reaches approximately 200 µV/K, which is higher than the value of approximately 140 µV/K observed for GeTe but slightly lower than the value of approximately 215 µV/K for GST. Figure 6b presents the temperature‐dependent *σ*. Relative to GeTe, Sb doping lowers *σ* from approximately 7000 to 925 S/cm at room temperature and from approximately 1500 to 800 S/cm at 793 K. In addition, further La doping leads to a slight increase in *σ*, with GST‐La reaching approximately 690 S/cm at 793 K and 990 S/cm at RT, compared with approximately 660 S/cm at 793 K and 925 S/cm at RT for GST. As shown in Figure 6c, GeTe, GST, and GST‐La exhibit comparable *PF* values of approximately 28 µW cm^−1^ K^−2^ at 793 K, while GST and GST‐La maintain higher *PF* values of 8 µW cm^−1^ K^−2^ at room temperature to 27 µW cm^−1^ K^−2^ at 673 K than GeTe from 5 µW cm^−1^ K^−2^ at room temperature to 25 µW cm^−1^ K^−2^ at 673 K. Although GST‐La exhibits a slightly lower *S* than GST, the corresponding increase in *σ* helps maintain a relatively high *PF*. Figure S2 shows the weighted mobility (*µ_W_*) of the samples. Sb doping significantly lowers *µ_W_* from 225 to 125 cm^2^ V^−1^ s^−1^, and La doping further reduces it to 120 cm^2^ V^−1^ s^−1^ at RT. This indicates the suppression of carrier transport at low temperature and the increase of DOS effective mass, resulting in the increase of *PF* at low temperature. When the temperature rises to over 573 K, *µ_W_* of the three samples are at the same scale, which is because acoustic phonon scattering becomes the dominant carrier scattering.

The temperature‐dependent *κ* is shown in Figure 6d. GeTe exhibits a relatively high *κ*, decreasing from approximately 7 W m^−1^ K^−1^ at room temperature to approximately 2.8 W m^−1^ K^−1^ at 793 K. Owing to the significant reduction in *σ* induced by Sb doping and the corresponding decrease in the electronic contribution to *κ*, *κ* is reduced to approximately 1.7 W m^−1^ K^−1^ over the measured temperature range for GST. Further La doping suppresses *κ* to approximately 0.8 W m^−1^ K^−1^, while GST‐La simultaneously exhibits a slightly higher *σ* than GST. Figure 6e presents *κ_L_*, obtained by subtracting *κ_e_* (Figure S4) from *κ*, where *κ_e_* was calculated using the Wiedemann–Franz law κ_*e*_ =  *L*σ*T*, where *L* is the Lorenz number. To account for the non‐parabolic band structure, the Lorenz number was calculated using the single Kane band (SKB) model [64] according to:

(2) $$L\;=1.1+1.35exp(\frac{-\left|S\right|}{[162-128exp(\frac{-Eg}{0.614})][\frac{15.71}{\sqrt{T}}+0.0925]})$$

where |*S*| is the absolute value of the Seebeck coefficient, *E*_g_ is the band gap energy, and *T* is the absolute temperature.

Compared with GST, *κ_L_* of GST‐La decreases from approximately 1.2 W m^−1^ K^−1^ at room temperature to approximately 0.20 W m^−1^ K^−1^ at 793 K, indicating enhanced phonon scattering. This reduction in *κ_L_* ​may be related to the combined effects of various structural features, including point defects and stacking faults associated with increased lattice disorder, as well as strain‐field fluctuations, domain and subdomain boundaries, planar defects, and microscale grain boundaries. These features may provide additional phonon‐scattering pathways and thereby contribute to the reduced *κ_L_*.

As a result, the substantial reduction in *κ*, together with the retained high *PF* and slightly improved *σ*, increases *zT* to 2.8 at 793 K, compared with 1.3 for GST and 0.7 for GeTe. Figure S1 in supply material shows the *zT* after several heating cycles. GST‐La sample maintains a *zT* over 2.5 after 8 cycles, showing high thermal stability.

## 3 Conclusion

GST‐La samples were synthesized by melting followed by spark plasma sintering, and their microstructure and thermoelectric transport properties were systematically evaluated. The incorporation of Sb effectively regulates the hole concentration and maintains the electrical transport properties, while reducing *κ_e_*. In addition, La doping enhances lattice disorder and introduces multiscale microstructural features, which significantly strengthen phonon scattering and thereby suppress *κ_L_*. The reduced *κ_L_* is associated with point defects, stacking faults, ridge‐like structures, domain boundaries, planar defects, and grain boundaries. As a result, the GST‐La composition achieves a peak *zT* of 2.8 at 793 K, together with a *κ_L_* of 0.8 W m^−1^ K^−1^ and a *PF* of 28 µW cm^−1^ K^−2^. These results highlight the effectiveness of combining composition tuning and multiscale defect engineering in improving the thermoelectric performance of GeTe‐based materials.

## 4 Experimental Methods

### 4.1 Synthesis

GeTe, Ge_0.9_Sb_0.1_Te, and Ge_0.89_Sb_0.1La0.01_Te samples were prepared via a melting method‐ annealing‐ spark plasma sintering (SPS) method. High‐purity elemental Ge (99.999%), Sb (99.999%), La (99.9%), and Te (99.9999%) were weighed according to their nominal compositions, loaded into evacuated quartz ampoules, and melted in a muffle furnace. The furnace was heated from room temperature to 1273 K for 12 h, held at this temperature for 10 h to ensure complete homogenization, then annealed at 873 K for 72 h, and finally cooled to room temperature in the furnace. The solidified ingots were manually pulverized in an agate mortar to obtain fine powders, which were subsequently consolidated into disks 12 mm in diameter by SPS under 50 mPa at 823 K. The SPS cycle involved ramping to 773 K in 10 min, then to 823 K in 5 min, and finally holding at 823 K for 3 min. The sintered Ge_0.89_Sb_0.1_La_0.01_Te pellet has a density of 5.874 g/cm^3^ calculated from the measured mass and the geometrical dimensions of each specimen, corresponding to a relative density exceeding 95% (Table S1).

### 4.2 Characterization

The phases of the samples were determined using X‐ray diffraction (XRD; D8 Advance powder XRD, Bruker) and differential scanning calorimetry (DSC; Q100, TA Instruments). The synchrotron powder X‐ray diffraction patterns were collected using the Powder Diffraction beamline (Australian Synchrotron, ANSTO, Clayton, Victoria, Australia). The micro‐ and nanostructures of the samples were investigated using scanning electron microscopy (SEM; JSM‐IT800, JEOL), and field‐emission transmission electron microscopy (HF5000 TEM, Hitachi).

### 4.3 TE Transport Properties

*σ* and *S* were measured simultaneously using the standard four‐probe method in a nitrogen atmosphere (SBA 467 HT, Netzsch). The *κ* was calculated by *κ* = *λC*_p_ *d*, where the thermal diffusivity (*λ*) is obtained through the flash diffusivity method (LFA 458 HyperFlash, Netzsch), specific heat (*C*_p_) is determined from the reported paper [65], and the density (*d*) is obtained by calculation, respectively. *κ* and *zT* calculated based on different *C*_p_ are shown in Figure S3.

### 4.4 DFT Calculation

The structure optimization and energy band structure are calculated within the framework of DFT using the generalized gradient approximation (GGA) [66] Perdew‐Burke‐Ernzerhof (PBE) exchange and correlation functional [67]. The pseudopotential projector augmented wave (PAW) method [68] is implemented in the Vienna Ab initio Simulation Package (VASP) [69]. The electronic structures of both the low‐temperature rhombohedral GeTe (space group R3m) and the high‐temperature cubic GeTe (space group Fm3̄m) phases were investigated. For Sb‐doped compositions, 3 Ge atoms were substituted by Sb, corresponding to the intended stoichiometries. For the Sb, La co‐doped compositions, 3 Ge atoms were replaced by Sb and one Ge atom was replaced by La, not exactly match the nominal experimental concentration. The deviation in La concentration arises from the finite size of the computational supercell and the requirement of maintaining a periodic substitutional model. The lattice parameters and atomic coordination numbers were fully relaxed under the following conditions: the residual force for ion convergence was 2 × 10^−2^ eV/Å, and the total energy converged to 10^−7^ eV/Å. The cut‐off energy of the plane‐wave basis set was 550 eV. Brillouin zone integrations were sampled on a Γ‐centered Monkhorst−Pack 5 × 5 × 5 mesh of k‐points in the Brillouin Zone. We calculated the electron spectrum at Bloch wave vectors along high‐symmetry lines (Γ—M—K—Γ—Z—L—H—Z) for rhombohedral GeTe and (X—W—L—Γ—K—Γ—L—W—X) for cubic GeTe in the Brillouin Zone.

## Author Contributions

**Ting Lu**: conceptualization, methodology, formal analysis, data curation, writing – original draft. **Huangshui Ma**: writing – review and editing, visualization, methodology, resources. **Mingquan Li**: writing – review and editing, validation, investigation. **Min Hong**: writing – review and editing, writing – original draft, funding acquisition, visualization, project administration, formal analysis, supervision, data curation, software.

## Conflicts of Interest

The authors declare no conflicts of interest.

## Supporting information




**Supporting File**: advs77821‐sup‐0001‐SuppMat.docx.

## Data Availability

The data that support the findings of this study are available from the corresponding author upon reasonable request.
