# Supplementary material for: Coordinated Electronic‐Transport Regulation and Multiscale Phonon Scattering in Sb–La Co‐Doped GeTe
Source: Adv Sci (Weinh). 2026 Sep 27:e77821. Online ahead of print. doi: 10.1002/advs.77821 (PMC13616346; doi:10.1002/advs.77821)
Supplement: Supplementary file 1 [file ADVS-9999-e77821-s001.docx]

Supporting information

**Coordinated electronic-transport regulation and multiscale phonon scattering in Sb–La co-doped GeTe**

*Ting Lu, Huangshui Ma, Mingquan Li,^a^ Min Hong,^*^*

Ting Lu, Huangshui Ma, Mingquan Li, Min Hong

Centre for Future Materials, School of Science, Engineering and Digital Technologies, University of Southern Queensland, Springfield, Queensland 4300, Australia

Email: min.hong@unisq.edu.au

1. Figure S1 shows the TE stability and repeatability of GST-La sample. After 8 cycles, the sample still have a *zT* over 2.5.

Figure S1. *σ* (a) and *S* (b) of GST-La sample at the 1,2,3,6,7 and 8 heating cycles.

2. Figure S2 shows the calculated weighted mobility (*μ*_W_) of the samples, which was determined using the following equation:

$$\text{μ}_{\text{W}}\text{=331(}\frac{\text{mΩcm}}{\text{ρ}}\text{)}{\text{(}\frac{\text{T}}{\text{300 }\text{K}}\text{)}}^{\text{-3/2}}\text{[}\frac{\text{exp}\text{[}\frac{\left| \text{S} \right|}{\text{k}_{\text{B}}\text{/}\text{e}}\text{-2]}}{\text{1+}\text{exp}\text{[-5}\frac{\left| \text{S} \right|}{\text{k}_{\text{B}}\text{/}\text{e}}\text{-1]}}\text{+}\frac{\frac{\text{3}}{\text{π}^{\text{2}}}\frac{\left| \text{S} \right|}{\text{k}_{\text{B}}\text{/}\text{e}}}{\text{1+}\text{exp}\text{[5(}\frac{\left| \text{S} \right|}{\text{k}_{\text{B}}\text{/}\text{e}}\text{-1)]}}\text{]}$$

Where *ρ* is the electrical resistivity, , *k*_B_is the Boltzmann constant, and e is the elementary charge. *μ*_w_ denotes the weighted mobility.^1^

Figure S2. Calculated weighted mobility of the pristine and doped samples.

3. The *κ* and *zT*of GST-La calculated based on different *C_p_* from report*_._* All data show low *κ* and high *zT* over 2.5 at 793 K. The *κ* and *zT* values of GST-La in this work were calculated using the experimentally measured *C_p_* of Ge_0.9_Sb_0.1_Te from report, which has a similar composition to our samples and yields a high *zT* of 2.8. To evaluate the sensitivity of the calculated *κ* and *zT* to the choice of *C_p_* , alternative *C_p_* values from other report were also used, and the corresponding results are presented in Figure S3. The calculated *zT* exceeds 3.0 when the Dulong–Petit value, the *C_p_* of GeTe, or the *C_p_* of Ge_0.86_Bi_0.04_Pb_0.1_Te is used. Using the *C_p_* of GeTe_1-_*_x_*Se*_x_* gives a *zT* close to 4.0. The *zT* values calculated from the *C_p_* data of (GeTe)_1-_*_x_*(In_2_Te_3_)*_x_* and Ge_1-_*_x_*Bi/Sb*_x_*Te are slightly lower, but remain above 2.6 at 793 K. These results demonstrate that the calculated *zT* is sensitive to the selected *C_p_* dataset, while the high *zT* of GST is consistently maintained.

Figure S3. The *κ* (a) and *zT* (b) of GST-La sample calculated based on different *C_p_* from report.^2^

4. Thermal diffusive, *L*, and *κ_e_*

Figure S4. Thermal diffusive (a), *L* (b), and *κ_e_* (c) of the pristine and doped samples.

5. Table S1 shows the calculated density and relative density

Table S1 The calculated densities and relative densities of the pristine and doped samples. The relative densities are calculated base on a theoretical density = 6.14 g cm^-3^.

| Sample | Calculated density (g cm^-3^) | Relative density (%) |
| --- | --- | --- |
| GeTe | 5.894 | 95.99 |
| Ge_0.9_Sb_0.1_Te | 5.898 | 96.06 |
| Ge_0.89_Sb_0.1_La_0.01_Te | 5.874 | 95.67 |

6. Figure S5 and Table S2 show the SEM results of GeTe sample. The element percentage indicates the existence of Ge impurities.

Figure S5. SEM image and related EDS of GeTe sample.

Table S2. The element percentage of GeTe sample from EDS result.

| Element | Mass (%) | Atom (%) |
| --- | --- | --- |
| Ge | 33.53 | 46.99 |
| Te | 66.47 | 53.07 |

7. Figure S6 and Table S3 show the SEM results of GST sample. The EDS map shows one Sb_2_Te_3_ impurity. The element percentage indicates the existence of Ge impurities.

Figure S6. SEM image and related EDS of GST sample.

Table S3. The element percentage of GST sample from EDS result.

| Element | Mass (%) | Atom (%) |
| --- | --- | --- |
| Ge | 27.15 | 39.48 |
| Sb | 6.60 | 5.72 |
| Te | 66.24 | 58.80 |

8. The element percentage of GST-La sample

Table S4. The element percentage of GST-La sample from EDS result in Figure 3a.

| Element | Mass (%) | Atom (%) |
| --- | --- | --- |
| Ge | 22.71 | 33.92 |
| Sb | 9.57 | 8.53 |
| Te | 67.72 | 57.55 |
| La | null | null |

**References**

1.	G. J. Snyder, A. H. Snyder, M. Wood, R. Gurunathan, B. H. Snyder, C. Niu, “Weighted Mobility,” Advanced Materials 32, no. 25 (2020): e2001537. <https://doi.org/10.1002/adma.202001537>

2.	J. Li, X. Zhang, Z. Chen, S. Lin, W. Li, J. Shen, I. T. Witting, A. Faghaninia, Y. Chen, A. Jain, L. Chen, G. J. Snyder, Y. Pei, “Low-Symmetry Rhombohedral GeTe Thermoelectrics,” Joule 2, no. 5 (2018): 976. <https://doi.org/https://doi.org/10.1016/j.joule.2018.02.016>
